# Revisiting the Miller-Kanazawa Debate: Should Asia Be Afforded More Attention From Evolutionary Psychologists?

**DOI:** 10.3389/fpsyg.2019.00547

**Published:** 2019-03-22

**Authors:** Sean T. H. Lee

**Affiliations:** School of Social Sciences, Singapore Management University, Singapore, Singapore

**Keywords:** Geoffrey Miller, Satoshi Kanazawa, debate, Asia, future of evolutionary psychology, response, opinion

A decade ago, Dr. Satoshi Kanazawa and Dr. Geoffrey Miller, two renowned evolutionary psychologists, participated in a debate on whether evolutionary psychologists should focus on Asia in their quest to establish a stronghold in the general field of psychology (Kanazawa, 2006; Miller, 2006a,b). Despite painstaking efforts to foster greater receptiveness among Western psychologists toward an evolutionary perspective of psychological phenomena, most still seem to be critically reserved toward it; favoring instead proximate sociocultural explanations of psychological phenomena as stipulated by the standard social science model (Tooby and Cosmides, 1992; Popper, 2003; Derksen, 2005). Facing such an inertia, Miller (2006b) suggested that it may perhaps be wise to re-direct the field's efforts on the up-and-coming Asia region, to potentially secure better prospects for the field of evolutionary psychology. Kanazawa (2006), however, opposed such a notion and expressed considerable doubt on whether Asia would be worth the effort.

Since then, much to Miller's (2006a,b) foresight, Asia has been exerting increasing impact on the field of psychology, churning out research that are more autonomous and distinctive than ever before; the trouble is, such growth has most notably been observed in the domain of social psychology—the flag-bearer of the standard social science model (Kitayama, 2007; Haslam and Kashima, 2010). On the other hand, with Miller's impetus stifled, the greatest effort made upon the region, since the debate, has arguably been the holding of one HBES (Human Behavior and Evolution Society) annual conference (out of 13 since then) in Asia (Japan). Without the consensual adoption of Miller's impetus among evolutionary psychologists, it is conceivable that Asia may, in time, arrive at a similar situation to its Western counterpart given its current trajectory, rendering it no less difficult to propagate and advance the field of evolutionary psychology to the general psychology community in the region, than in Western regions. Before that happens, it is perhaps timely to reconsider if Asia is truly not worth the effort, as proposed by Kanazawa (2006). In this paper, key contentious issues raised during the debate pertaining to the pragmatism of Asians, the creativity of Asians, and language barriers will be discussed, along with the musing of relevant sociopolitical considerations.

## Pragmatism of Asians

Miller (2006a) stated that Asians' inclination toward the study of majors that are more pragmatic, such as management and the hard sciences in-general, and their overall “bias toward hard sciences” are key impediments to evolutionary psychology propagation (p. 116). While it may be true that Asians are particularly drawn to STEM (Science, Technology, Engineering, and Mathematics) fields (Chien, 2011), such a proclivity may not necessarily be an impediment. On the contrary, it may even render evolutionary psychology propagation in the region an easier endeavor.

To elaborate, the core precept of evolutionary psychology is that all psychological phenomena, like biological phenomena, operate under an overarching, guiding theory—the theory of evolution (Ghiselin, 1973). This is in stark contrast to the predominant standard social science model, wherein a slew of dynamic processes, such as socialization and enculturation, rather than a single overarching theory, guide psychological research and practice; making an evolutionary perspective a tough pill to swallow for many Western psychologists (Cosmides and Tooby, 1997; Staats, 1999). STEM fields are, however, generally guided by certain overarching theories and frameworks. For instance, quantum theory and Einstein's theory of relativity, guide physics-related practice and research (Brown, 1987), while the theory of evolution itself guides research and practice in the biological sciences (Dobzhansky, 2013). As such, increased receptivity toward STEM field majors may, instead, advantageously translate into greater receptivity toward evolutionary psychology, due to potentially greater appreciation of having an overarching theory to guide research and practice.

To appeal to Asians' pragmatism, Miller (2006a) also suggested that “evolutionary versions of educational, industrial, organizational, consumer, and political psychology” (p. 118) can be produced and promoted to Asian psychologists. However, this may not play to the strengths of evolutionary psychology, which specializes in ultimate rather than proximate explanations of psychological phenomena (Scott-Phillips et al., 2011). Miller's (2006a) suggestion may inadvertently require the examination of proximal factors and causes that are presently well-detailed within the standard social science model. Specifically, instead of examining how one's behavior may have arose due to certain adaptive advantages it conferred during ancestral times, we may have to consider how certain factors in the immediate environment may have precipitated the behavior in question, so that we can reliably predict and potentially exert control over such a behavior. This may, in turn, lead Asian psychologists back to the other domains of psychology that adopt the standard social science model (e.g., social psychology), wherein a wealth of frameworks and predictive models for such proximal processes already exists.

I believe that the successful propagation and advancement of evolutionary psychology requires embedding the perspective of evolution within all distinct domains of psychology as opposed to pushing its adoption as a distinct domain or an alternative perspective of psychology. Take the field of biological science for example; few biologists would ever explicitly talk about the theory of evolution (let alone try to prove it) when explicating biological phenomena housed under distinct domains, such as genetic drift (genetics) or the development of antibiotic resistance among bacteria (medical microbiology); such phenomena are automatically expounded upon under the precepts of evolutionary theory (Allendorf, 1986; Berkowitz, 1995). Arguably, this should be the ultimate goal of evolutionary psychology and Asia, with its marked pragmatism and proclivity toward STEM fields, might just be the region conducive enough for the pursuit of such a goal.

## Creativity of Asians

Kanazawa (2006), while noting that Asians possess generally high levels of intelligence, cautioned that their dismal levels of creativity is of a huge concern. Specifically, Kanazawa (2006) posited that Asians' general deficiency in creativity precludes them from producing innovative and impactful research, rendering the region a less-than-ideal option for the propagation and advancement of evolutionary psychology. While this assertion has sparked some level of public outcry (e.g., Farmer, 2011), there are, however, some kernels of truth to this.

Though results of studies that have administered the Kirton's Adaptation-Innovation Inventory on Asian samples suggest that Asia may not be as deficient in innovative individuals as depicted (e.g., Ee et al., 2007), studies have shown that Asians tend to possess lower levels of psychological traits predictive of exceptional scientific accomplishments (e.g., inquisitiveness) and that this seems to stem from genetic bases (e.g., Kura et al., 2015). This is in addition to the studies cited by Kanazawa (2006) showing how environmental factors specific to Asia, such as its educational systems, may serve to discourage creative thinking. Nonetheless, this discussion fundamentally draws its roots from the sociobiological debate on nature vs. nurture, rendering the question on to what extent do such genetic predispositions predetermine Asians' academic creativity and to what extent might the modulation of environmental factors improve their academic creativity, an empirical one that deserves to be further examined (Mackinnon, 1962; Baer, 1978).

Regardless, being adept at incremental research as opposed to radical “scientific revolutions” (Kanazawa, 2006, p.123) may not necessarily be a drawback. One should be quick to note that the overprizing of novel, “revolutionary” research and the slighting of incremental research is a large contributory factor toward the current replication crisis in the general field of psychology to begin with (Giner-Sorolla, 2012; Nosek et al., 2012). As such, it may actually be advantageous for the field of evolutionary psychology to have a large community of Asian researchers churning out incremental research, gradually advancing our knowledge of evolutionary psychology by producing work that, while not revolutionary by any means, are markedly robust and replicable.

## Language Barrier of Asians

Kanazawa (2006) also noted that Asians' “inability to express themselves in English is likely to hamper Asians' contribution to evolutionary psychology” (p. 122). Admittedly, with English generally being the international language of scientific communication (Ferguson et al., 2011), low proficiency of the language may potentially impede one's ability to propagate his/her ideas within the international scientific community, especially in the context of scientific conference presentations. However, they may still be able to contribute significantly via written publications. In addition to the existence of journals specifically aimed at translating manuscripts written in non-English languages to English, such as Psychological Science (China), many reputable international journals, such as those published under the American Psychological Association (APA), offer seamless English language editing services in collaboration with third-party service providers. The use of such channels potentially allows Asian psychologists who may not be as proficient in English to still be able to convey their ideas and findings to the international scientific community.

As noted by Miller (2006a), many founding psychologists were Germans who overcame Eurocentrism and ported their work to the United States. Despite English not being their first language, these forebearers still managed to contribute significantly toward the general field of psychology. Arguably, one's ability to express his/her ideas in a clear and cogent manner may be a more determining factor of potential scientific contribution as opposed to the language in which it was conveyed in. Admittedly, though, this is by-large an empirical question that requires further examination. Nonetheless, with increasing learning and mastery rates of the English language within the regions of Asia (Hu and McKay, 2012; Kirkpatrick, 2012), this language barrier issue is expected to become less of a concern over time.

## Sociopolitical Orientation

Beyond these concerns raised by Dr. Miller and Dr. Kanazawa, another key factor that should be considered is one's sociopolitical orientation. In a study conducted by Buss and von Hippel (2018), it was found that most Western social psychologists hold a left (i.e., liberal) sociopolitical orientation and, as such, strongly endorse a *tabula rasa* view of human nature that flies squarely against the precept of evolutionary psychology, which stipulates that humans possess heritable predispositions. Comparatively speaking, Asia holds a much more conservative sociopolitical orientation in general, which is possibly indicative of greater receptiveness toward evolutionary psychology (Aspalter, 2001).

It is worth noting, however, that there seems to be increased ideological liberalism in the region over recent years, especially among the affluent (Holliday, 2000; Goodman et al., 2013). It is therefore possible that such potential receptivity may decline over time and that more Asian psychologists may subscribe religiously to the standard social science model. This is already evinced in studies showing yearly increases in proliferation of social psychology research in the region (e.g., Haslam and Kashima, 2010), suggesting that time may be running out for evolutionary psychology propagation in the region.

## Conclusion

Notwithstanding potential language issues that may wane with time, Asia appears to be a region with great potential for the propagation of evolutionary psychology. The region's general proclivity toward the hard sciences, potential for producing robust and replicable research, and generally conservative sociopolitical orientation renders it a potentially lucrative stronghold for evolutionary psychology. However, with changing sociopolitical orientation and increasing adoption of the standard social science model, its viability may soon dissipate. Greater attention and effort from Western evolutionary psychology communities, such as organizing more conferences in this region or proactively taking up more faculty positions in this region (even as visiting faculty), is therefore urgently needed, before it is too late.

A decade has already passed since Miller's prudent nudges. In another decade's time, when we look at Asia again, will we observe the successful permeation of evolutionary psychology, or will we observe high levels of inertia toward the adoption of an evolutionary perspective of psychology as we currently observe in the West?

## Author Contributions

The author confirms being the sole contributor of this work and has approved it for publication.

### Conflict of Interest Statement

The author declares that the research was conducted in the absence of any commercial or financial relationships that could be construed as a potential conflict of interest.
